# Foreign

**Published:** 1841-07-17

**Authors:** 


### FOREIGN.

*Case of Amputation of the Neck of the Womb followed by Pregnancy; with remarks on the Pathology and Radical Treatment of the Cauliflower Excrescence from the Os Uteri.* By JAMES Y. SIMPSON, M. D., Professor of Midwifery in the University of Edinburgh.—In his learned work on the Diseases of Females, (Dublin, 1838,) Dr. Churchill remarks, (p. 249,) "I am not aware that any attempts have been made in Great Britain to exercise the *cervix uteri*."

The following instance of this operation may therefore not be uninteresting, either as regards its details, or the hitherto flattering success that has resulted from it.

In the beginning of May last I was requested by Dr. Lewins of Leith, to visit with him Mrs. Cameron, who, as he informed me, had a tumour attached to the *cervix uteri*.

The patient, aged 33, had been married for thirteen years. During that period she had borne five living children, and suffered from a miscarriage at the sixth month. In June 1838, she weaned her youngest child. For about a month previously to that date she had a red discharge from the vagina, which was constant in its occurrence, though not great in its quantity. It continued during the autumn. In October, she passed with labour pains of three or four hours duration, a body which the midwife in attendance supposed to be an abortion of the second month. During the period of pregnancy with this alleged abortion, the vaginal discharge was still present. It increased considerably after October, and was now often mixed with coagula of blood. It had always a very offensive smell and more or less of a red tint, but sometimes it appeared comparatively pale and watery. The discharge was as profuse though less discoloured during the night, and when at rest, as during the day and when taking free exercise. From the supposed period of abortion in October, up to the period that I saw her with Dr. Lewins in May, three or four cloths were soaked regularly every twenty-four hours by it. Whenever she ventured to walk about without napkins she felt the discharge "running" (to use her own expression) from her. On two separate occasions the escape of pure blood became suddenly so great as to pass through all the cloths and create great alarm. Mrs. C. was not aware of any causes which excited these attacks of hæmorrhage. One of them occurred during the night. She never observed any monthly increase in the discharge answering to the catamenial periods.

During the whole course of the disease Mrs. C. had not suffered (if we except the temporary expulsive uterine action in October) any pain or uneasiness whatever in the region of the uterus; but by the time that I first saw her she had become greatly weakened and reduced by the abundant discharges. Her face was pale and anæmic, and she was occasionally obliged to keep her bed in consequence of exhaustion.

Dr. Lewins was first called in to see the patient a short time previously to my visiting her along with him. On examination *per vaginam* I found, as Dr. Lewins had described to me, a tumour fixed to the posterior lip of the uterus. It was then about the size of a small pear, and was attached by a very broad basis. The surface of the tumour felt somewhat rugged and granulated. It was firm but not hard in its consistence. The patient did not complain of any pain upon touching or pressing its surface. Its superficial vessels bled freely under every attempt at examination. On introducing the *speculum vaginæ*, and embracing the diseased mass within the further extremity of the instrument the surface of the tumour was seen to be irregular, and of a bright-red, strawberry colour.*

* It is almost unnecessary, we believe, to insist at the present day, upon the importance of the early and accurate local examination of the uterus in all cases of suspicious vaginal discharges. In some instances, examination by the finger may be sufficient, but in every doubtful case the speculum should likewise be resorted to if there is any affection of the vagina or cervix. We have found it often confirming, and not unfrequently also changing and rectifying the opinion which the mere tactile examination had led us to adopt. In this country great difficulties have been placed against the more general introduction of the speculum into practice in consequence of the disagreeable and revolting exposure of the person of the patient, which is usually considered necessary in its employment. We have latterly in our practice endeavoured to avoid this very natural objection, by teaching ourselves to introduce and use the instrument when the patient was placed on her left side in the position usually assumed in making the tactile examination, and with the nates near the edge of the bed. We strongly recommend our professional brethren to follow this plan, as by it, and with attention to the management of the bed-clothes, we have found that the instrument can be perfectly employed with little, or indeed without any exposure of the body of the patient. The speculum is introduced easily without the assistance of sight, and the mouth of it only requires to be afterwards uncovered, in order to enable us to examine the *cervix uteri* and top of the vagina. We have made trials of many different forms of specula, and find, for almost all purposes, that of Ricord by far the most manageable. In exposing the *cervix uteri* for the purpose of drawing blood from it by scarifications, in cases of chronic congestion and metritis, we have occasionally employed a tubular speculum with advantage, but even in this case the double-bladed instrument is equally useful, and in some instances preferable. In a case of ulcer of the *os uteri* which we are at present attending with Dr. John Gairdner, and where the passages are much relaxed and the uterus very low in the vagina, we have, on Dr. Gairdner's suggestion, employed with much advantage a short tubular speculum of only an inch and a-half in length, and with a deficiency or opening along the course of one side of it, of sufficient size to enable us to pass our finger for the purpose of placing the diseased part in the proper centre of the instrument. We have thus been enabled to touch easily the ulcerated surface with different applications; while with the usual instruments it was found a very difficult task to fix in this instance the very mobile *cervix uteri*.

It appeared possible to grasp the basis of the tumour with a ligature ; but both Dr. Lewins and I were of opinion that the free amputation of the *cervix uteri*, with the deceased structure attached to it, offered by far the most probable means of success. We communicated this opinion to the patient's husband, and at the same time stated, that, even under this method of treatment, the disease would probably recur. After a delay of about three weeks, Mrs. C. announced that she was ready to submit to the operation that we had proposed. In making a re-examination after that short interval, I was perfectly convinced that the excrescence had grown considerably, and was extended in its base so as to involve more of the angles of the *os uteri*, as well as of its posterior lip.

On the 25th of May, I proceeded to excise the *cervix uteri*, and was assisted in the operation by Dr. Lewins and Mr. Ziegler.

The patient was laid upon her face, her body placed across the bed, and her lower extremities allowed to hang over the front of it. The thighs were held separate from one another. My object was to pull down the diseased neck of the uterus until it protruded externally beyond the mouth of the vagina, and then freely excise it. For this purpose I introduced the two first fingers of my left hand into the vaginal canal up as far as the tumour, and used them as a guide by which I fixed the teeth of a long vulcellum into the sides of the excrescence, its tissue, however, was so soft as to tear under slight traction, and thus afford me little purchase for pulling the mass downwards. The instrument was refixed nearer the root of the excrescence, and a second vulcellum was superadded to render the purchase the more secure. With these I was enabled to pull down the tumour gradually and cautiously until it was entirely protruded beyond the external parts. Dr. Lewins and Mr. Zeigler having satisfied themselves that the *cervix uteri* and whole bulk of the tumour was extruded, I cut off the protruded mass, dividing it from behind forwards, and removing the whole vaginal portion of the *cervix uteri*. The uterus immediately slipped up into its natural position. Very little hæmorrhage followed. I stuffed, however, the vagina pretty firmly, under the fear that dangerous bleeding might supervene.

The patient bore the operation well, and complained wonderfully little during it. In the evening, Dr. Lewins had to remove the vaginal plug, in order to allow her to evacuate the bladder. It was not considered necessary to replace it. No marked morbid symptoms whatever, either local or constitutional, followed.—The great vaginal discharge immediately ceased. On being interrogated in relation to this point on the second day, the patient emphatically described herself to Dr. Lewins as "*quite dry*," and that for the first time for many months. The incised surface, when examined through the speculum a few days after the operation, presented a healthy granulating appearance. It was not considered advisable to allow her to sit up till the tenth day after the operation was performed, and in a few days more she began to walk about the house and perform her usual domestic duties.

She has not been one hour sick since the period of the operation, and has now regained her usual strength and spirits.

No morbid discharge from the vagina of any kind has hitherto appeared. She has never since menstruated; and about five weeks ago she fancied that she felt the symptoms of quickening. On examining the abdomen to-day (14th November) with the stethoscope, I heard distinctly both the placental souffle and the sounds of the fœtal heart. The *os uteri* is closed, and on examination by the finger, gives the sensation of a firm puckered cicatrix.

The excrescence after its removal was found to measure two inches and three quarters at its broadest part, and two inches and a quarter at its greatest depth. The thickness of it, where it implicated the posterior lip of the *os uteri* was one and three-eighths of an inch, but on either side it stretched forward, and involved the angle between the anterior and posterior lips; thus rendering this admeasurement greater on its lateral parts. The anterior lip of the *os uteri*, which was fully removed as high as the reflection of the vagina, seemed sound except at the above angles. The posterior surface of the posterior lip was densely and completely covered by the excrescence, up to the reflection upon the vagina. In excising the diseased part, I removed it so high as to bring away all around, a small portion of the reflection itself of the mucous membrane of the vagina. The surface of this portion of membrane, as thus removed in attachment to the upper edge of the excrescence, appeared quite healthy on careful examination of the excised mass. The surface of the tumor presented a well-marked small granulated appearance, with deeper fissures crossing it, and giving it an irregular and lobulated appearance. The sides of it were considerably and deeply lacerated in various places by the teeth of the vulcellum. On rubbing down any small part of the recent tumor between the finger and thumb, a kind of vascular or cellular frame-work was all that was left behind. The mass, before dividing it, was steeped in a strong alcoholic solution of corrosive sublimate, in order to insure its preservation. On making a section of the tumor after it had been thus sufficiently indurated, it presented to the touch and sight an appearance greatly resembling that of the brain when hardened by the same menstruum. A number of minute cells are scattered over the surface of the section. On slightly rubbing any part of the section, but particularly the more external part of it with the handle of the scalpel, its apparently homogeneous structure at once breaks up and revolves itself into an immense number of very small connected grape-like granules. These same granules impart to the external surface of the excrescence its peculiar minutely mammillated structure; while their arrangement into nodules, in consequence of the divided and lobulated arrangement of the superficies of the tumor, gives to the whole a striking resemblance to the head of the cauliflower.

On submitting some very thin slices from the surface of the section of the tumor, to a powerful microscope in the possession of Dr. Reid, it was seen to be composed of a number of cells, arranged in some places in groups, in others in irregular lines. These cells contained each a large nucleus, and this nucleus inclosed several small nucleoli. The structure in question of cells or cystoblasts, incasing nuclei and nucleoli, has been shown to be so common as an elementary form of natural structure, by Schleiden and Schwann, and as an elementary form of various morbid tissue by Valentin, Gluge, and Müller, that no conclusion, in the present state of our knowledge, can be positively drawn from this microscopic structure alone. But it may be interesting to add, that none of the caudate or spindle-shaped bodies described by Müller as often existing in morbid encephaloid structures were seen in any section that was examined.

*Pathological Nature of Cauliflower Excrescence.*—The history, symptoms, physical characters, and minute structure of the preceding tumor appear to refer it indubitably to that species of growth which was first accurately distinguished and described by Dr. Clarke, under the quaint but expressive name of the "Cauliflower Excrescence from the *os uteri*."*

The pathological nature of this variety of morbid growth has given rise to considerable difference of opinion among physicians. Drs. Gooch, Hooper, Davis, and Lee, regard it as truly cancerous in its character. Others, as Drs. Clarke, Burns, and Waller, regard it as a morbid tissue, not necessarily of a malignant or carcinomatous nature. A number of circumstances appear to me to show, that in reference to at least the first stage of cauliflower excrescence, the opinion of these latter authors is probably correct. The occurrence of the disease in some cases as early as the 20th year of life;† its occasional shrinking and almost total disappearance upon the application of a ligature, or after death;‡ the frequent slowness of its general progress during life; the apparent absence of diseased deposits in the neighboring tissues and parts upon the dead body;§ and, above all, the alleged restriction and even complete removal of the tumor, in one or two instances, by the use of astringent applications and other simple means,* form so many circumstances strongly pointing to the opinion that in the earlier part of its progress the tumor cannot be regarded as of a carcinomatous character.

Has it any analogy in its pathological nature and origin—as it certainly has in its physical characters—with the soft warts and condylomata that sometimes form on the mucous membrane of the vulva and entrance of the vagina? These warts and condylomata have the same tendency to regeneration after their imperfect removal, and present to us a striking exception to the general pathological law of the local reproduction of a morbid growth being a sign of its malignity.

But, whatever view we may take of the primary nature of the cauliflower excrescence of the *cervix uteri*, we have sufficient evidence for believing either that this disease has been often confounded with carcinomatous or medullary fungus from the *cervix uteri*, from the want of adequate diagnostic marks to distinguish them; or that, though non-malignant in its commencement, the cauliflower excrescence may, like some other local benign growths, become the seat of carcinomatous deposit and malignant action, during its progress. Thus it has been found by Gooch† and Madame Boivin‡ to return again in a malignant form, after its imperfect removal by the ligature or knife. In an instance mentioned by Dr. Davis,§ its removal was followed, after the lapse of a considerable period, by its reproduction, and ultimately by carcinomatous ulceration; and in two cases that occurred to Professor D'Outrepont|| and Siebold,¶ in which large tumors, having a cauliflower form were found affixed to the *cervix uteri* during parturition, the neighboring uterine tissues, as well as the contiguous structures of the bladder and uterus, were found in a carcinomatous state upon the *post mortem* dissection.

---

* Transactions of a Society for the Improvement of Medical and Chirurgical Knowledge, Vol. iii. p. 321. (1809.)

† Sir C. M. Clarke on the diseases of Females, Vol. ii. p. 62.

‡ Ibid p. 70 and 75.

§ Ibid p. 66 and 70.

---

* Ibid p. 105 and 108. A lady, aged 32, had a continued profuse watery discharge, mixed occasionally with blood. She was greatly weakened, pale and emaciated. A cauliflower mass projected from the surface of the *os uteri*. Several remedies, with cupping and local astringents, were ordered and assiduously persevered in. After two years, "*no difference* could be felt between the *os uteri* of the patient and that of a woman in perfect health." (Clarke, p. 107.)

† On the most important Diseases peculiar to Women, p. 288.

‡ Heming's translation of Boivin and Duges' work, p. 300.

§ Principles of Obstetric Medicine, Vol. ii. p. 744.

|| Abhandlungen Geburtshulflichen Inhalts, Th. i. p. 275.

¶ Dissertatio sistens casum singularem carcinomatis uteri cum graviditate conjuncti.

In another case, in which Michaelis* excised what he terms a *fungus medullaris* with a cauliflower appearance, from the anterior lip of the uterus during labor, the posterior lip of the organ afterwards degenerated, and cancer of the stomach ultimately supervened.

If these latter cases were not merely more advanced stages of the cauliflower excrescence, but, as appears to us not improbable, diseases originally and pathologically different from it, though resembling true cauliflower excrescence in its peculiar form and external physical characters, are there any means which might enable us to form a diagnosis between the two affections? The whole subject is one certainly demanding more careful observation and deeper investigation. The nature and characters, both physical and chemical, of the vaginal discharges in these and other maladies of the sexual parts, require to be more accurately examined and discriminated. May the degree of mobility of the *cervix uteri* serve in any case as a source of diagnosis? "The tendency of cancer, (as observed by Müller,†) is to interfere with the natural structure of surrounding parts, while those formations which are of a benignant nature leave the neighbouring healthy tissues unaltered." In carcinoma of the *cervix uteri*, we thus generally find, at even a pretty early stage of the disease, that the organ has become more *fixed* and immoveable than natural, in consequence of the morbid deposit affecting both the structure of the neck of the organ and the contiguous surrounding tissues. Does the reverse of this hold good with regard to cauliflower excrescence of the *cervix uteri*?

*Radical Treatment of Cauliflower Excrescence.*—Different measures have been proposed for the radical removal of cauliflower excrescences from the *cervix uteri*. The caustic, ligature, and knife, have each been employed.—With regard to the two former it seems superfluous to hope that the good results following upon their use can be more than temporary. The basis of the diseased structure will in all probability be left. Occasionally both the caustic and the ligature appeared to have produced injury rather than good, by the irritation and increased action that they have excited in the diseased parts.

If any radical operation and cure for cauliflower excrescence be attempted, the excision of the tumour with the whole of the vaginal portion of the *cervix uteri*, to which it is attached as a basis, appears to us to be the only measure which can at all be hoped to insure ultimate success. The disease has no doubt recurred in repeated instances even after this operation. In some of these cases it probably had advanced too far onwards to a carcinomatous character. In others the failure might be attributable (as confessed by Boivin and Duges, in regard to the cases which they themselves report,) to "the tumour being alone removed," and not the *cervix uteri* also, which forms its seat, and "is always more or less affected."* In a few authenticated cases on record, in which complete amputation of the cervix uteri with the attached tumour was performed, the patient was known to have remained free from any symptoms of the disease for several years afterwards. A search through the medical literature of the last twenty years would, in all probability enable us to adduce several such instances; but it may be sufficient for our present purpose to adduce three cases, of which we have notes lying before us, and that appear to us, as far as we can judge from the details and expressions of the reporters, to have been probable instances of the same species of tumour that Dr. Clarke originally described.

CASE 1.—In an instance of what is termed fungus cancer (*cancer fongeux*) by Colombat,† that surgeon amputated the *cervix uteri* on the 2d June, 1830. The wound completely cicatrized, and the patient's health was re-established. She died in April, 1832, of epidemic cholera.

The fungus cancer, Colombat observes in another part of his work, (p. 711,) is one of the forms of cancer which is the least liable to return after excision of the parts.

CASE 2.—Boivin and Duges mention a case of cauliflower excrescence of more than two inches in diameter, which was attached to the anterior lip of the *cervix uteri*. It was removed, along with more than six lines of the *cervix uteri*, in November, 1828. The patient was alive in October, 1832, and is then reported by the above authors as only labouring under some symptom of the menorrhagia and dysmenorrhœa at menstrual periods.‡

CASE 3.—An instance is reported by Duparcque, under the head of "Exuberance de l'Uterus," in which Herviz de Chegoin excised the two lips of the uterus, affected with what the operator terms "a granular strawberry inflammation," and which he alleges has often been confounded with cancer. The discharge and other symptoms of the disease had been present two years previously to the operation. At the date of the report (four years after the excision of the diseased part) the patient remained perfectly well.

In the case of Mrs. C. which I have above reported, I undertook the amputation of the diseased part with, as has already been said,

* Neue Zeitschrift fuer Geburtskunde, Bd. iv. S. 176.

† On the Nature and Structural Characteristics of Cancer, West's translation, p. 66.

* Heming's Translation, p. 301.

† Colombat de l'Isere; Traite des Maladies des Femes, Tom. ii. p. 701.

‡ See Heming's Translation of Boivin and Duges' Treatise on Diseases of the Uterus, p. 300–301, and drawings of the excrescence in the Atlas, Pl. xxiv.

strong doubts as to its ultimate success. The patient's peace of mind was broken, and her constitution was so rapidly breaking down under the constant, profuse, and weakening discharges which afflicted her, that she would in all probability have soon sunken under them. Immediately after the operation was performed these discharges completely ceased, and have never since returned. Her health and strength have been in the mean time restored to her; and she is at the present moment, as I have already shown, advanced beyond the middle period of pregnancy. The morbid characters of the diseased structure that I removed are such certainly as to render its future regeneration not at all improbable; but as yet there are no local appearances of its return; and,—taking the very worst view of the case,—there seems to be no reasonable doubt but that the operation has restored the bodily comfort, and prolonged the life of the patient, if it has not entirely freed her from the risk of a future return of the disease.—*Edinburgh Med. and Surg. Jour.*

---

*Report of the Labor of a Patient in whom the neck of the Uterus was Amputated.* By Robert Lewins, Junior, M. D.—From the 14th November until the 19th December, Mrs. C. remained in good health; but on that day she was attacked by violent pain in the abdomen, which occasioned alarm, and rendered it expedient to have recourse to blood-letting, and other active measures, by which the urgent symptoms were speedily removed. From this period until February 15th, she remained quite well. On the evening of that day, my father was requested to visit her. He found the membranes ruptured; and that there had been a considerable discharge of *liquor amnii*, tinged with blood. On making an examination, the uterine aperture was found to be as large as a sixpence; the parietes of the organ, as far as the finger could reach, being very thin, tense and rigid. The presentation of the child was ascertained to be natural. No labor pains had as yet come on; nor did they commence during the subsequent night. About half-past ten on Tuesday evening, however, (16th February,) they became pretty smart and regular, and from this period she may be considered to have been fairly in labor. On being summoned about two o'clock on Wednesday morning, I found the opening into the uterus about the size of a shilling, of an ovel shape, the long diameter of which was in a line leading from the right iliac synchondrosis to the left acetabulum; slight oozing of the *liquor amnii* was still taking place, notwithstanding which, the bag of membranes distended with fluid, was protruded in front of the child's head during every pain. This I could only account for by supposing that the place at which it had given way had been displaced from the situation it had formerly occupied opposite the aperture in the uterus, before all the fluid had been evacuated. The edges of the *os uteri* were very rigid, sharp, and tense, conveying to the finger the sensation of a tendinous ring, upon which the labour pains, although very regular, produced little or no effect in the way of dilatation.—Matters remaining in precisely the same condition at five A. M., I had recourse to artificial dilatation of the *os uteri*, which was persevered in cautiously, and at intervals during the pains, with the very best effect. Considerable progress had been made at eight o'clock, when the uterine orifice was as large as a half-crown piece; the edges, however, still continued so rigid and tense, that during every pain I feared laceration would occur.

Since manual interference had been resorted to, the pains had been violent; and about nine o'clock A. M., a feeling of cramp being complained of in the abdomen, during the absence of the ordinary uterine contractions, I took away some blood from the arm, which was followed by immediate and complete relief. Dilatation was continued at intervals throughout the forenoon, and very slow and gradual progress was made with the labour, the patient complaining greatly of pains in the back and thighs. On several occasions during, and immediately after attempts at dilatation had been made, she had a fit of vomiting, which was also excited on her assuming the sitting posture. During all this time she preferred remaining upon her back—her sufferings appearing to be more supportable in that position than in any other. The vagina and *os uteri* were well lubricated with axunge. Early in the afternoon the pains assumed more of an expulsive character, the anterior edge of the uterine aperture being protruded, rigid, and unyielding in front of the child's head into the vagina. About half-past three, the pains increased in severity and duration, but no further dilatation of this portion of the uterus took place. A few minutes before four, however, I succeeded in slipping this impediment over the child's head, which immediately afterwards, during a prolonged pain, was expelled from the *os externum.* The remainder of the process went on without difficulty. The infant seemed to have suffered somewhat from pressure, but, upon being sprinkled with cold water, it completely revived. Soon after the birth of the child, a pain expelled the placenta into the vagina, from whence it was easily removed; the whole process being thus completed in seventeen and a half hours from the first accession of regular labour pains.

The child was healthy and vigorous, and although unquestionably before the full time, the operation of excision having been performed on the 25th of May last, presents no marked deviations from a nine months' child, its weight being seven pounds, and its length being 19½ inches. Mrs. C. was troubled with severe after-pains, for the relief of which an opiate draught was prescribed, in consequence of which she enjoyed a good night's rest, and on the following morning was fully as well as she usually is on such occasions.

Her progress since has been uninterruptedly favorable; her convalescence was rapid; she is at present in the enjoyment of perfect health, and quite fit for her duty as a nurse.

In the annals of medicine there are few cases recorded which afford more striking and gratifying proof of the triumph of our art over a disease which, from its nature and seat, had by British surgeons been considered desperate and beyond the reach of human aid.

There is, however, reason to suppose that the illustrious Ambrose Paré contemplated the possibility of performing this bold operation; and Lauvariol, a French surgeon, actually proposed it in 1780. We find Wrisberg, Schlessing, and Krevel in Germany, and Monteggia in Italy, soon afterwards advocating the propriety of excising the *cervix uteri;* but there is no evidence of its having been performed on the continent until 1801, when it appears to have been successfully practised by Osiander, Professor of Midwifery in the University of Gottingen; and in a paper communicated by Dr. Thomas, our present distinguished Professor of Pathology, and published in the number of this Journal for July 1816, there is a minute and interesting statement of Osiander's mode of operating in nine successful cases.

Soon after the publication of Osiander's paper, my father, then a young practitioner, suggested to the late Mr. Welsh, of Haddington, the expediency of an attempt to excise the *cervix uteri* in the case of a lady of high rank, who labored under malignant disease of that organ—a proposal which at that time was considered, in Scotland, preposterous, or something worse. Osiander informs us that, according to the testimony of all who have submitted to this operation, it is not near so painful as one might imagine. Other continental operators have expressed themselves to the same effect; and this fact was also verified in the case of Mrs. C.

Dupuytren, Recamier, Colombat de l'Isère, and Lisfranc, are the principal French surgeons who have distinguished themselves as operators in this department. The latter asserts that he has performed the operation of section of the womb ninety-nine times; the accuracy of that statement, however, has been called in question by his countryman and ex-premier prosecteur, M. H. Pauly, who nevertheless admits that Lisfranc was eminently successful in one case, that of Madame Carpentier, which forms, according to him, "la gloire vivante de l'amputation du col de l'uterus, c'est elle que depuis son operation a eu quatres enfans dont deux jumeaux"—a case which, to the honor of Scottish surgery, now no longer stands unrivalled.

With Drs. Churchill and Simpson, I am ignorant of any attempt which has been made in Great Britain before the present case to excise the *cervix uteri.* Dr. Davis, it is true, in his learned worked on the Principles and Practice of Obstetric Medicine, refers somewhat vaguely to its having been performed with at least temporary success in England. In 1741, (just one hundred years ago,) Mr. Pugh of Chelmsford removed with perfect success during parturition, a large "fleshy excrescence that grew on the edge of the *os tincae.*" (See his Treatise of Midwifery, p. 122.) Mr. Bell extirpated a polypus from the same locality during labor, (Edin. Med. and Surg. Jour. Vol. xvi.;) and Levret, Gooch, &c., have given drawings and cases illustrative of such pediculated polypi and excrescences of the cervix. Professor Syme and others have removed tumors of the same nature from the unimpregnated *os uteri;* but these I conceive to have been very different operations from excision of the neck and part of the body of the womb.

The successful result of the cases referred to have demonstrated an important physiological fact, that the presence of the cervix and *os uteri* are not indispensable to the existence and due consummation of pregnancy. Lisfranc, indeed, remarks that labor is more easy after the removal of these parts; but in this he is assuredly mistaken. The cicatrized condition of the uterine aperture is calculated to render its dilatation exceedingly tardy and painful; and in our case continued artificial assistance was required to accomplish the object, generally easily effected by the unaided efforts of nature; and, notwithstanding this artificial aid, the first stage of labor was not completed until after a lapse of upwards of seventeen hours, although the patient had previously born five children, her last labor having only lasted two hours.

In another point our experience does not coincide with the representations of Lisfranc, who informs us that the wound of the uterus is not favorably circumstanced for quick cicatrization, to promote which, he recommends first, injections of emollient, afterwards of stimulating fluids, and subsequently cauterization with the liquid protonitrate of mercury. The incised surface in Mrs. C.'s case, when examined through the speculum a few days after the operation, presented a healthy granulating appearance; and it is certain that conception took place within ten days from the date of the operation.

In conclusion, I would earnestly insist on the importance of early and accurate examination of the uterus by the speculum in all cases of suspicious vaginal discharges. The result in Mrs. C.'s case is to be attributed to the precise nature of the disease being promptly ascertained. Cases of a similar nature are, I fear, frequently treated by the routine practitioner as simple menorrhagia; whilst the malady is insidiously gaining ground until the performance of an operation no longer affords a rational prospect of relief. The ill-judged opposition to the employment of the speculum has contributed to create, and confirm, a prejudice against the use of that instrument, which may probably, in some measure, explain why we are deficient, when compared with our continental brethren, in the knowledge and treatment of uterine diseases; a reproach which the precepts and example of Professor Simpson, as triumphantly manifested in the result of Mrs. C.'s case, will, I trust, tend materially to remove.

P. S.—Since writing the above remarks, I have been favored by Dr. Simpson with the perusal of a communication to him from Dr. Ingleby of Birmingham, in which that distinguished accoucheur relates two interesting cases in which he performed the operation under consideration. "I once amputated," says Dr. Ingleby, "the *cervix uteri* for cauliflower growth. It was unaccompanied by pain, as these cases always are. Hæmorrhage, serous discharge, dropsy of the extremities and face, with general anæmia, were the prominent signs. All the disease was removed which was connected with the uterus. Small bits, however, grew from the mucous membrane of the vagina. Whether caustic would have succeeded in eradicating these, I am unable to determine, as thoracic inflammation came on a few days subsequent to the operation, and the patient died from it and the effects of a very large vomica in one lung. Every part of the body was sound except the lungs and the mucous membrane of the vagina, just below the cut surface of the *cervix uteri*, and opposite the *os* and vaginal portion of the organ. I once also excised the *cervix uteri* for a bleeding cancerous fungus, which did not extend above the *os uteri* more than a quarter of an inch. I removed all the disease, and likewise some of the sound parts. The patient, who was almost moribund prior to the operation, became apparently quite well, got actually fat, and remained in good health for a year. The disease then returned, chiefly in the vagina and bladder, in consequence of which she died."

Dr. Ingleby also relates, cursorily, a case in which he witnessed this operation performed, without, however, arresting the progress of this dreadful disease.—*Ibid.*

---

*Transactions of the Liverpool Medical Institution.*—Mr. Long related the following case of *Tubercle in the Cerebellum*:—

Whilst attending a lady, in February last, I was requested to see a servant who was suffering from sick headache; she had nausea, and occasional slight vomiting; her bowels were constipated, and tongue slightly furred; a disagreeable bitter taste in the mouth; pain in the occipital region extending to the vertex, and a perfectly quiet pulse.

I ordered an aperient, which evacuated the bowels freely, and then directed small doses of blue pill to be taken at bed-time, and a mild aperient each morning. In a few days the tongue became perfectly clean and moist; the disagreeable taste had disappeared; but the vomiting and headache persisted, having still the characters of that form of headache usually denominated sick headache.

I ordered her to bed, with the intention of examining her more carefully. I found the abdomen quite soft, without the slightest degree of tenderness in the epigastric region, or elsewhere. I then inquired into the state of the uterine system; her age was forty; the catamenia were regular as to time, and healthy in quantity and quality. She informed me that for upwards of a dozen years she had, at intervals, suffered from attacks similar to that which she now experienced, and that at times the pain in her head was so severe as to confine her to bed; that these attacks frequently came on at the menstrual period, but, if they occurred prior to it, were always aggravated by it; that at first they were easily removed by a brisk aperient, but had progressively become more obstinate; and that the attack under which she was then laboring had existed a fortnight before I saw her.

She described the headaches as commencing usually during the night, or towards morning, and when very severe being always attended with vomiting, which occurred most frequently after eating, but frequently without any assignable cause; in fact, it was the obstinacy of the vomiting which drew my attention more particularly to the case; for medicines and treatment directed by this particular symptom were of no use whatever.

Taking into account the healthiness of the uterine functions, the quietness of the pulse, the natural state of the tongue, the freedom from pain or tenderness in the epigastric region, and the persistence of the vomiting and periodical headache, I felt little doubt but that these phenomena owed their origin to the cerebro-spinal system; in effect, on making pressure between the occiput and atlas she experienced an acute pain extending from this region to the vertex, and immediately vomited.

I directed leeches to be applied below the occiput; they produced marked relief. I repeated them, and the headache and vomiting ceased; she slept soundly during the night, which she had not done for weeks before, and was able to go about the house as usual. At this period I did not remark any peculiarity in her gait, except that she moved about cautiously, as if afraid of shaking her head.

In a few days the symptoms began to return. I directed a blister to be applied to the same region. This checked them at once; she improved, was able to go out, and stated that although she did not feel quite well, yet that she was in her usual state of health. In this state she continued, and at my urgent solicitation applied a second blister, but could not be prevailed upon (being relieved from her distressing symptoms) to persist in the treatment I had laid down for her.

Towards the end of March she went into the country, and consulted a practitioner there, who was of opinion that all her ailments resulted from a "change of life," and that the leeches and blisters had been injurious. He recommended some medicines, which she took; the effect, however, not bearing out the opinion she had received, but her headache and vomiting recurring, she returned to Liverpool, and I was requested to see her in consultation with a physician of this town. I found her much worse. The headache and vomiting were as bad as at first, with occasional hiccup, and now and then slight difficulty of deglutition. In attempting to walk she staggered like a drunken person, and supported herself by holding a chair, or placing one hand against the wall. In sitting down she was some time before she could steady herself. At times partial convulsive movements occurred. These were peculiar; the muscles at the back of the neck first became rigid, the head being drawn backwards, and then twisted to the right side, and then convulsive movements of the muscles of the face on the same side commenced. These convulsive movements were sometimes accompanied by a tremor of the whole body. The menstrual secretion was still regular and healthy; the tongue quite clean, but tremulous; the bowels easily moved by medicine; the pulse slow, but otherwise natural; the temperature of the body below the natural standard.

The plan of treatment adopted included slight irritation of the nape of the neck by means of lunar caustic, and she continued under our care until the middle of May, the convulsive twitchings increasing in severity, the headache, unsteadiness in walking, and vomiting persisting. It is worthy of remark that the convulsive twitchings and accompanying phenomena had for the last week or two assumed an intermittent character, so that she had what she called a good day and a bad day; the good day was only so, however, when compared with her bad day. This always commenced with headache during the night, then vomiting, then convulsive twitchings of the neck and right side of the face, and then a tremor of the whole body. During her bad day she was confined to bed; during her good day she sat up or walked about in the unsteady manner I have described. She was once seen by one of the family, when attempting to walk in the garden, to roll completely over. Her sister also informed me, and was corroborated in her statement by her fellow-servants, that she had frequently a tendency to fall forwards, and would have done so had not she or they caught her.

She went to reside with her sister in town from this period until the evening of the 28th May (about a week.) I heard nothing of her. On the evening in question I was sent for, and found her seated in a chair, dead. She appeared to be asleep, so that I had some difficulty in persuading her friends that she was actually dead. Her sister informed me that during the preceding week she had been rather worse than usual; that the convulsive twitchings had extended to both arms, both being equally affected; that, on the morning of the day on which she died, she felt better than usual, but expressed a strong conviction that she should not recover. In the evening her headache commenced, the convulsive twitchings succeeded, she complained of sickness, a basin was held before her, she opened her mouth as if to vomit, and expired. She had passed her usual catamenial period eight days.

I examined the body eighteen hours after death, and was assisted by Mr. Ellison. There were no external signs of scrofula. Nothing unusual was found in the membranes of the brain, or in its substance. A considerable quantity of colourless fluid escaped from the lateral ventricles; the arachnoid investment of both choroid plexuses was considerably distended by fluid underneath, presenting a hydatid-like appearance. The pia mater investing the cerebellum was much more minutely injected than could be accounted for by the effects of mere gravitation, particularly as the pia mater investing the posterior lobes of the brain was not similarly injected. The external configuration of the cerebellum was natural. Its whole substance and surface were rather softer than usual. The inferior vermiform process was occupied by a tubercle the size of a common marble, of a somewhat irregular figure, extending about a quarter of an inch into the right lobe. The nervous tissue immediately surrounding it was softer than the rest of the cerebellum. The superior surface of the right restiform body, just where it plunges into the cerebellum, was much softer than the left. The medulla oblongata, the pons varolii, and the nerves, arising from them were perfectly healthy. We were not permitted to examine into the state of the spinal cord, or any other part of the body. The tubercle was hard, and when cut into presented granules of concrete pus, and appeared to me to be contained in a fine cyst.

*Summary of the symptoms.*—Senses unaffected; intellectual faculties entire, except that the memory seemed at times impaired; catamenia regular, and no derangement of sexual functions; periodical headache extending from the occiput to the vertex, of twelve years' standing.

Pain on pressing the occipital region; vomiting without epigastric tenderness; clean but tremulous tongue; occasionally slight difficulty of deglutition; rigidity of the muscles of the neck, with convulsive twitchings drawing the head to the right side; twitchings of the muscles of the right side of the face, extending subsequently to both arms; want of command over the lower limbs, with tendency to fall forwards.

Lesions found after death: tubercle in the inferior vermiform process of the cerebellum, extending into the right lobe; softening of the cerebellum, particularly around the tubercle; injection of the pia mater investing the cerebellum; softening of the right restiform body; fluid in the lateral ventricles of the brain.

1. The points worthy of notice are the rarity of tubercles of the cerebellum. Thus Louis and W. Lombard found tubercles in this organ twice only in 450 tubercular adults.

2. The length of time these productions may exist without giving rise to serious symptoms: a case is mentioned by Dr. Abercrombie in which a tubercle of the cerebellum evidently dated its commencement at least five years prior to death.

3. The intermittent phenomena caused or induced by a permanent change of structure, or new production, depending probably upon the intermittence of the lesions which exist around it; and, consequently, the existence of a new production may give rise to no phenomena, so long as the surrounding tissues are unaffected by it, and remain in a state of integrity.

4. The non-appearance of the menstrual discharge at its usual period—eight days prior to death; the aggravation and extension of the symptoms during this period; the existence of a considerable quantity of serum in the lateral ventricles: though a question may arise whether death was caused by the vessels relieving themselves by this effusion, or whether it was produced by syncope.

5. The softening of the right restiform body, and the remark of Rolando, "That injury of one of the restiform bodies produced convulsions, with curving of the body of the animal to the injured side;" there being in this particular a coincidence.

6. The want of accordance between the case I have related and the case related by Mr. Serres, which induced him to place the seat of sexual impulse in the middle portion of the cerebellum (on this point I was particularly anxious, and purposely delayed its consideration for this place.) Eighteen years ago she had a son, who is now alive; since that period she has had no intimate male acquaintance, although she has not shunned their society; and during the whole of my attendance I did not perceive the least symptom of any amorous propensity, and her sister assured me that since her mishap she is certain she had none.

I may mention here that Mr. Montault has elated a case of a tubercle an inch in size occupying the whole vertical thickness of the middle portion of the cerebellum. The individual was paraplegic, and addicted to women; but in this case there existed other disease of the brain, and the lumbar vertebræ were diseased; whereas M. Guerard mentions a case where a tubercle an inch and a half in size existed at the upper surface of the cerebellum, and in the middle line; the substance of the cerebellum around the tubercle to the extent of two lines was softened; the spinal cord was sound. This individual was weak, particularly in his lower limbs, and staggered in walking. It is added it would be impossible to say that there was any marked symptom referring to the genital organs. The case rather seems to favour the opinion of M. Magendie, who, it is stated in the Journal Hebdomadaire, ascertained, by direct experiment, that when an injury was inflicted upon the middle portion of the cerebellum the animal remained undecided in its movements; or considering the cerebellum as a whole, without reference to its middle portion, it seems to favour the opinion of M. Flourens and M. Bouillaud, that in it resides the faculty of combination of the movements; and coincides with the cases related by Lallemand, Gall, and Guerard, the individual staggering in walking, and having a tendency to fall forwards.

7. The accordance of the symptoms in the case I have related with the cases of disease of the cerebellum related by Andral, these being thirty-six cases of disease of various kinds, and of variable extent, and eleven cases of abscess.

Thus we find the intellectual faculties affected in 5; the senses in 6; the motor powers in 30; the tongue, in thirty-six cases, 2; headache in 35; vomiting in 17.

With respect to derangements of the motor powers they are stated to have been various; but amongst them we find several in which paralysis affected especially the lower limbs; whilst it is noted, however, that involuntary contractions of a greater or less number of muscles is a more common phenomenon than paralysis: in a great number all the body was agitated by convulsive movements, at intervals, in other particular muscles, as those of the neck, drawing the head backwards, or to one side. He also mentions the facts stated above, that some individuals had a sort of uncertain gait, staggering like a drunken person, and having a tendency to fall forwards.

I may here mention two cases, one by Andral. Convulsive movements occurred, and always commenced by a powerful agitation of the head, which was drawn backwards as in a variety of tetanus. Some days the convulsions were confined there; but at other times became more general, and almost all the muscles of the body were affected: they augmented in frequency and intensity, extended to the respiratory muscles, and the patient died in a sort of asphyxia. An encysted abscess, the size of a pullet's egg, existed in the left lobe of the cerebellum. The second by M. Recamier. Frequent convulsive movements existed, commencing always in the muscles of the neck. In these attacks all the body was agitated, and the head drawn backwards. An encysted abscess, the size of a pullet's egg, existed in the left lobe of the cerebellum.

With respect to the headache, it is remarked by Andral that it was generally seated in the occipital region; was mostly intense, and in several cases assumed a periodical or intermittent character; and that when vomiting occurred it was in all sympathetic, and one of the predominant symptoms, not occurring as a simple complication, but being certainly connected with the disease of the cerebellum.

Dr. Abercrombie remarks, that where periodical headaches and paroxysms of vomiting occur in indurations of the nervous centres, the prominent morbid appearances are found in the cerebellum, and that the uneasiness in the head is more permanent and fixed than we should expect to find in a dyspeptic case, and the uneasiness is increased by causes which would probably be beneficial in dyspeptic headache; such as activity and cheerful company.

He also notices that in cases of paraplegia tumors and indurations often exist in the cerebellum or tuber annulare; but that the cases on this point are unsatisfactory, in consequence of attention not having been paid to the spinal cord.

Diseases of the central organs of the nervous system are involved in great obscurity, and must be so so long as we are ignorant of the functions of their separate parts, so long as we have a difficulty in ascertaining the precise amount of a lesion, or of conveying to another a precise idea of it when ascertained; for a degree more or less of an injury may make a vast difference in the amount or kind of symptoms produced; or a disorganization or change of structure in a part endowed with a special function may, according to its extent or locality in that organ, in one case alter, in another, weaken and destroy its functions, or call into action another part with which it may have a direct or secret influence. I think, however, that sufficient may be gathered from the above remarks to enable us, in certain cases, if not to diagnosticate diseases of the cerebellum, at least to enable us to state with some degree of probability that disease of this organ does exist.—*Lon. Med. Gaz.*

---

### ABERDEEN INFIRMARY REPORTS.

*Cases and Observations by* —— LAING, Esq., one of the Surgeons of the Hospital.

*Case of Cystic or Hydatoid Disease of the Testis.*—A. M. æt. 29, admitted April 11th, 1838, with a large swelling of the left side of the scrotum. The tumor is puriform, somewhat elastic, and has an indistinct fluctuation, but by no means the feel of hydrocele. The cord seems to be sound. He says the swelling began several years ago, without any known cause, commencing towards the lower part of the scrotum. The opposite testis is considerably enlarged and hard. On examination a cicatrix was observed at the lower part of the scrotum, which he says was the result of an abscess which occurred there about two years ago, and burst spontaneously, but soon healed. It burst again last winter, and discharged for several weeks. His complexion is florid; but his constitution is feeble, and seemingly scrofulous. A calomel pill was ordered, followed by Epsom salts, and a cooling lotion to relieve the superficial inflammation, which seemed to have been excited by the journey.

April 19th.—An operation having been determined on, an incision was cautiously made through the integuments of the scrotum and tunica vaginalis, when a bluish semitransparent membrane presented itself. Into this a small trocar was cautiously pushed, but only about half an ounce of transparent serum escaped. On withdrawing the instrument another similar membrane appeared, which was drawn outwards with dissecting forceps, and punctured, and found to contain only two or three drachms of serum. In this manner many similar cysts were successively drawn forward and opened, to the number of upwards of thirty, as the quantity of serum amounted to sixteen ounces. The wound was then closed with adhesive plaster.

Two days after the operation he was seized with febrile symptoms, which were treated by saline and antimonial medicines; and this was followed by erysipelas of the face: the pulse rose to 125, and he had much confusion and delirium. The head was shaved, cold lotions applied, and a band one inch broad made between the face and the scalp by the nitrate of silver. These means, with purgatives, saline and antimonial medicines, subdued the fever and the erysipelas of the face; but the wound in the scrotum was still considerably inflamed.

Adhibeantur Cataplasmata.

30th.—He is now convalescent, pulse 84; tongue clean; the wound of the scrotum discharging healthy pus.

May 12th.—The opening in the scrotum contracting, and the discharge much diminished. The testis of the left side is now little above the natural size, though the scrotum is still thickened and enlarged. The right testis is still hard and somewhat painful to the touch.

23d.—Dismissed.

This patient returned to the hospital in July, 1839, on account of a different disease. The testis formerly operated on was found quite sound, being scarcely larger, but somewhat softer than natural, without any pain. This was evidently a case of the disease called by Mr. Guthrie, and others, cystic or hydatoid disease of the testis, but differed from most of the cases recorded in the size of the cysts, which are usually described as being from the size of peas to that of grapes.

—

*Case of Diseased Testis.*—E. A. ætat. 35, wright, was admitted on the 9th of January, 1840, with a large tense elastic swelling of the left side of the scrotum, neither affected by coughing nor change of position, but extending up the cord as far as the abdominal aperture. It gave him little uneasiness, unless what arose from its bulk. On the whole the symptoms closely resembled those of hydrocele, but the tumour was not transparent, and the fluctuation was less distinct. It commenced about twelve months ago, without any known cause, and extended gradually upwards. On the 13th an incision was cautiously made through the integuments and the tunica vaginalis, when a hydatid of considerable size presented itself. This was punctured, and was immediately followed by another, which was also punctured, exactly as described in the preceding case; and thus ten or twelve ounces of serum were evacuated. As soon as the first cyst appeared, I sent for two of my colleagues, and they were witnesses to successive puncturing of the cysts. When the scrotum was reduced nearly to the natural size, the edges of the wound were brought together with adhesive plasters.

14th.—The wound continued for some time to ooze out a very watery fluid. To-day the scrotum is slightly swollen and inflamed.

Adhibeantur Cataplasmata. Low diet.

15th.—Was seized with severe pain of the testicles soon after yesterday's visit. The scrotum is now much enlarged, red, tense, and extremely tender; pulse frequent; tongue furred, and white; bowels opened by medicine this morning.

℞ Antimon. Tartar. gr. iv.; Aquæ Cass. ℥viij.; Solve Capiat. unciam, tertiis horis. Continuentur Cataplasmata et Fotus.

18th.—Swelling still great, but pain diminished. The integuments have assumed a gangrenous appearance in several places. Four or five incisions were made into the scrotum, which discharged blood and serum.

Continuenter Omnia.

20th.—No sloughing has taken place on the left side of the scrotum, the matter having found vent by the incisions; but a large slough four or five inches in length, by two in breadth, has formed on the right side.

Omittatur Mistura. Continuenter Cætera. ℞ Opii gr. i., formâ Pilulæ, omni nocte.

23d.—The slough, which comprehends almost the whole part of the scrotum, is now detached, and the right testis threatens to protrude. It is retained by adhesive straps. The febrile symptoms have recently subsided.

He was ordered porter and Sulphuric Acid mixture. Continuenter Pilulæ Opii bis die, et Oleum Ricini pro re nata.

February 10th.—Health improved, and strength returning rapidly. The ulcer is granulating and cicatrizing steadily. The testis of the affected side is reduced nearly to its natural size. Pergat.

18th.—Going on extremely well. The ulcer is diminished to the size of a crown-piece.

Omittantur Pilulæ Opii.

March 10th.—The wound has continued to contract gradually, and is now healed. The cicatrix is extremely small, compared with the great size of the ulcer. Dismissed cured.

Although the treatment adopted in these two cases proved successful, yet the symptoms were so severe, and the cure so tedious, that I doubt whether extirpation of the testis would not be preferable.

—

*Case of Secondary Hæmorrhage cured by Ligature of the Femoral Artery.*—J. C. æt. 24, a pilot, was admitted on the 22d of April, 1838, with both legs severely fractured. The accident was occasioned by his legs being entangled in a rope attached to a ship in motion. The left leg had suffered a compound and comminuted fracture, the tibia protruding through the wound. The bones of the right leg were comminuted, but did not protrude. There was, however, an external wound, and much contusion of the soft parts. There was also considerable hæmorrhage from the wound of the left leg, which was, however, soon checked by cloths soaked in cold water. It being determined to attempt saving both legs, they were set as accurately as possible, and splints and bandages applied. An anodyne draught was ordered at night, and the usual treatment in all other respects.

On the 25th the wound of the right leg had assumed a gangrenous appearance, which rendered it necessary to remove the splints from it, and apply poultices, fomentations, &c. The wound of the left leg was looking well. It was dressed, and the splints re-appplied.

On the 5th of May a large portion of the tibia was extracted from the wound of the right leg, which soon after began to assume a more favourable appearance; but, about the 10th, the left leg and knee were attacked with phlegmonous erysipelas, and diffuse abscesses formed near each malleolus. These were opened by the lancet, and discharged profusely. His strength was now supported by nourishing food, wine, porter, &c., and the wound of the right leg began to granulate favourably; but the abscesses in the left leg discharged copiously, and the heel began to slough from pressure. He was also affected with irregular fever and occasional diarrhœa, for which astringents, opiates, and quinine, were employed.

In the month of July the ulcers in the right leg were nearly cicatrized, and the bones had begun to reunite: but the bones of the left leg remained loose and bare, the sloughing of the heel continued, the os calcis became carious, and his strength was so much reduced by the discharge that it became necessary, in the beginning of August, to propose amputation of that limb, in order to save his life. The operation was accordingly performed on the 14th, at the usual distance below the knee, a flap being formed from the calf by transfixion. On the 20th the stump looked well, union by the first intention having taken place to a considerable extent. On the night of the 24th copious hæmorrhage occurred, in consequence of his having started up in bed from sudden alarm. It was arrested by cold and pressure. On the 25th it recurred with great violence. On taking off the dressings the lips of the wound were found to be forced asunder by coagulated blood. On clearing it away, the state of the stump was found to be such that it was impossible to seize and tie the bleeding vessels. A piece of sponge was, therefore, placed over them, and pressure applied by means of compresses and bandages.

27th.—Hæmorrhage has returned with violence four or five times since yesterday. Each attack of bleeding is preceded by severe pain in the stump. His strength is now alarmingly reduced, the lips and caruncula lacrymalis being pale, the pulse 160, feeble, and undulating. Amputation above the knee being considered inadmissible from his great debility, I proceeded immediately, with the assistance of my colleague Dr. Dyce, to tie the femoral artery, as the only remaining alternative. The incision was made in the usual place, the inner edge of the sartorious muscle raised, the sheath of the vessels opened, and a single ligature passed round the artery. Fortunately not a teaspoonful of blood was lost during the operation, as the patient had very little to spare. The coagulated blood was now removed from the face of the stump, but no hæmorrhage followed. The flap was, therefore, re-applied, and the wound dressed. From eight to twelve hours after the operation the temperature of the limb was six or seven degrees lower than that of the opposite side, notwithstanding the application of warm bottles, flannel, &c. Next morning it was only two degrees lower, and in the course of the following day became quite natural.

On the 2d September, the pulse had fallen to 112, and was firmer, and his colour began to return. The wound in the fore-part of the thigh had adhered by the first intention, except where the ligature passed out. The stump also was granulating freely, and had begun to cicatrize. Wine, quinine, and nourishing food, were continued. From this time he continued to improve steadily.

On the 18th the ligature came away from the artery, and soon after the wound closed. The stump also was nearly skinned over, and his strength much restored.

On the 14th of October the stump was entirely healed, and the right leg firm, and but slightly deformed. He was soon after dismissed, quite well.

—

*Case of Delirium Traumaticum followed by Secondary Hæmorrhage four weeks after amputation.*—P. J., æt. 50, was brought to the hospital at 9 P. M. of the 22d of May, 1840, with compound fracture, and lacerated wound of the left leg, caused by a loaded cart passing over it. Both tibia and fibula were fractured in the lower third, and three or four inches of the tibia protruded. There was also another extensive wound above the malleolus externus: the tendo-achillis and great bloodvessels were divided, and the ankle-joint laid open. He had lost a great quantity of blood during a journey of five miles since he sustained the accident, and it continued to drain away from the very extensive wounded surfaces, in spite of the application of the tourniquet, and pressure over the femoral artery over the os pubis. It was therefore resolved to amputate immediately, as the only chance of saving his life. The operation was performed by forming a flap four or five inches below his knee. Very little additional blood was lost.

May 23d.—Had no sleep during the night, though he took a dose of morphia towards morning; he complains of extreme pain in the stump; pulse 120; tongue dry; skin hot. 9 P. M. has been extremely restless and incoherent during the day; tongue brown and dry; pulse 130; bowels not moved; stitches removed from the stump.

℞. Calomel, gr. v.; Opii. gr. i. formâ pilulæ horâ somni, et Ol. Ricini. ss. crasmane.

24th.—Is considerably better, and more collected to-day; pulse 110; tongue moister; bowels freely opened, but much thirst; stump dressed with two or three adhesive straps, and water dressing.

Habeat Haust. Efferves. Subinde.

27th.—Continues still feverish and uneasy; pulse 106; tongue moister. No adhesions have taken place, but the flap hangs loose and sloughy; it is supported by two or three straps, and a poultice applied. The effervescing draughts and occasional doses of castor-oil continued.

29th.—Delirium again came on yesterday afternoon, and increased to such violence during the night, that it was necessary to put on the straight jacket to prevent him getting out of bed, and making for the window; pulse 110; tongue not dry; bowels not moved for two days. The head was ordered to be shaved, and cloths dipped in cold water constantly applied, and a blister put between the shoulders. In consultation it was agreed to give calomel in doses of two grains every four hours.

31st.—Delirium continues, but the pulse is rather stronger. Pergat. 8 P. M. Much the same; has had no sleep for 36 hours.

℞. Tinct. Opii. gtt. xxxv.; Aq. Menth. ℥ss. M. Fiat haust.

June 1st.—Slept occasionally during the night, but is still very delirious; pulse 100; tongue foul; passes his urine unconsciously.

℞. Sol. Mur. Morph. gtts. xl., statim. Vin. Albi. Hispan. ℥iv.

9 P. M. Has slept a good deal, and is certainly quieter; perspiration abated; pulse 96, and more regular.

℞. Sol. Mur. Morphiæ gtts. xlv.

3d.—Has improved gradually since last report, and is now quite sensible: pulse 96. He takes 45 drops of Sol. Mur. Morphiæ every night, and 30 every morning. Is ordered four ounces of white wine daily, nourishing diet, and effervescing draught at pleasure.

8th.—Improving steadily; has omitted the morning dose of the Mur. of Morph. for two days. The wound is very large and open, but it is suppurating and granulating in a tolerably healthy manner. Pergat.

20th.—Considerable arterial hæmorrhage took place this morning, which, however, ceased on the application of pressure to the femoral artery, and a piece of lint dipped in creosote over the bleeding point.

22d.—Hæmorrhage recurred this morning, but was arrested by the same means as before.

23d.—Arterial hæmorrhage came on to an alarming extent during the night, and again this morning; it was found to proceed from three distant points of the stump. From the state of the parts it was in vain to think of tying the vessels; it was, therefore, resolved to tie the femoral artery, and this accordingly I immediately proceeded to do, with the assistance of my colleague, Dr. Keith. A small cutaneous branch, which was divided in making the first incision, was immediately secured. A single ligature was then passed round the artery, and the wound dressed as usual. Next day the temperature of the stump, which had sunk two or three degrees soon after the operation, had arisen to the natural standard; and the granulations, which had become somewhat livid, resumed their florid colour. The secretion of pus, which had been copious, gradually diminished from this time, and cicatrization went on steadily. The ligature came away from the artery on the 14th day, and the opening through which it passed soon closed. On the 20th of July the stump was nearly healed, and on the 10th of August he was dismissed cured.—*Ibid.*

---

*Musœ Volitantes*. By A. L. Wigan.—Having ascertained, beyond a possibility of doubt, the nature of this annoying defect of vision, a few words of explanation may relieve the anxiety of many a nervous patient, and also be satisfactory to the profession. It is possible that others have made similar observations; but if so, they have not been communicated to the public in any work that has ever attracted my notice. I have often heard even medical men express a dread of ultimate consequences from this simple, evanescent, and unimportant malady.

I received my first knowledge of this disease (if indeed it can be called a disease) from the celebrated oculist, Mr. Ware; and not doubting that a man so well acquainted with the organ of vision, its physiology and pathology, must be correct in his opinions, I made, during many years, futile attempts at cure, without investigating the matter for myself. Having, however, suffered annoyance in my own person from this cause, I was induced to examine it with more attention, and I then found that all the ingenious explanations which had been offered were absolutely without a shadow of foundation, and that the case was nothing more than an opacity of the lachrymal fluid.

To be convinced of this it is only necessary to fix the eyes steadily on some stationary object, and abstain from letting fall the eyelids. Chains of luminous rings, black spots, little brilliant annular specks, and other fantastic forms, gradually and slowly fall down in a sort of shower. The moment the lid is dipped into the groove conveying the tears to the punctum lachrymale, all these objects are dispersed into fresh forms, infinitely varied, to fall down again gradually, as before, over the eye, while kept steady, or to be spread right and left by the slightest lateral motion; the chains of luminous rings, more especially, being broken and re-arranged *ad infinitum*.

The conviction produced by this simple experiment is at once so complete and so instantaneous, that I never yet met with a man who required a single word to be added to confirm his belief; and when it is considered how minute a substance, so near the axis of vision, may produce such an effect, and how naturally different degrees of opacity and adhesiveness may give the form of black spots, annular specks of light (single or in chains,) &c., the phenomena are easily explained.

The causes of this malady are various; disordered stomach, fatigue, want of sleep, long fasting, and many others, may be enumerated, each of which must be addressed by its appropriate remedies; but in the immense majority of cases the disease is not connected with constitutional or general disturbance, but is completely local, and may be removed by a collyrium of sea water, or the application of a blister not larger than a sixpence near the lachrymal gland, and a collyrium composed of one grain of sulphate of quinine to three ounces of distilled water, with just sufficient excess of sulphuric acid to keep the fluid transparent.—*Ibid.*

